# The effects of diagnosis-related groups payment on efficiency of the hospital health care in Croatia

**DOI:** 10.3325/cmj.2021.62.561

**Published:** 2021-12

**Authors:** Karolina Kalanj, Rick Marshall, Karl Karol, Stjepan Orešković

**Affiliations:** 1 Department of Medical Oncology, Clinic of Oncology, Clinical Hospital Center Zagreb, Zagreb, Croatia; 2 Epidemiologist and independent consultant in health system funding models, Eaglehawk Neck, Tasmania, Australia; 3 Independant consultant, Melbourne, Victoria, Australia; 4 Univesity of Zagreb School of Medicine, Andrija Stampar School of Public Health, Zagreb, Croatia

## Abstract

**Aim:**

To assess the impact of Croatian reforms related to the funding of inpatient care on the efficiency of acute hospitals.

**Methods:**

Between 2009 and 2018, the study analyzed resourcing, performance, and financing data for 33 acute hospitals. It used data from the Croatian Health Insurance Fund (CHIF) and the Croatian Institute of Public Health and included hospital activity and diagnosis-related grouping; average length of stay (ALOS); hospital staffing; CHIF revenue streams; and hospital incomes and expenditures.

**Results:**

During the study period, the cost-efficiency of Croatian public hospitals did not meaningfully improve. While ALOS decreased by 14% and the number of beds decreased by 12%, bed occupancy rates decreased by 9%, acute inpatient admissions by 5%, and diagnosis-related group (DRG)-weighted output by 16%. Hospitals operated at higher costs, as the average cost per DRG-weighted case increased by 17%, from HRK 11 828 in 2016, to HRK 13 897 in 2018.

**Conclusions:**

In this period, Croatian reforms failed to improve hospital efficiency. This may be explained by the failure of reformers to heed the experience of other countries, which showed that hospital payment reform of this nature calls for systematic and coordinated actions, inter-agency collaboration, and a strategic approach where the various interventions are in congruence and act to reinforce one another.

In the mid-1990s, European countries took action to reform their health provider payment systems to better control health expenditures and improve the performance of their health sectors (1). This occurred at a time when Croatia was going through a period of transition and was struggling to meet the financial needs of hospitals, which threatened the financial sustainability of the health sector as a whole (2). In order to address this growing problem, Croatia embarked on hospital financing reforms. It followed international practice of replacing its historic budget hospital funding model with an output-based system. The reform began in 2002, when hospital activity payment criteria were introduced in the form of classifications called payment per therapeutic procedure (PPTP). The PPTP system comprised 116 broad payment groups representing high-volume inpatient cases, which accounted for a large proportion of hospitals’ inpatient expenditure. While well intended, the system proved ineffective due to flaws in both its pricing and the structure of the payment mechanism (3).

In 2007, Croatia procured a license for the Australian Refined Diagnosis Related Groups (AR-DRG) which like all DRGs, is primarily an inpatient classification system that groups patients into clinically meaningful and resource-use homogeneous groups. As DRGs measure the level of hospital inpatient activity, it is possible to gauge hospitals’ relative efficiency by relating each hospital’s resource use to its output as measured by DRG activity (4,5). The country embarked on DRG implementation with the following key stakeholders: the Ministry of Health (MOH), which has the stewardship role over the health system and is also the operator of the nation’s tertiary referral hospitals; the Croatian Health Insurance Fund (CHIF), a statutory agency responsible for the national mandatory and supplementary health insurance systems and the central national purchaser of health care services; and county governments and the City of Zagreb, which operate hospitals in their own regions.

The aim of this study was to assess the effectiveness of the reform in the funding of inpatient care that has involved the implementation of the DRG system.

## MATERIAL AND METHODS

The study analyzed retrospective data related to the financing and performance of Croatian hospitals funded by CHIF through the DRG system over a ten-year period from January 1, 2009 to December 31, 2018. DRG activity and financial data for 33 hospitals, which provide 96% of inpatient acute care by public hospitals in Croatia,^,^ were made available by CHIF, while hospital resourcing data were obtained from the Croatian Institute of Public Health.

Hospital financial data included information on levels of State and local government funding; other income including patient co-payments; and hospital expenditure on staff, utilities, drugs, maintenance, food, and other items.

Croatian DRG coding practice for episodes of care uses a mix of International Classifications of Diseases Australian Modifications (ICD-10AM) and International Classifications of Diseases (ICD-10) for diagnosis, and the Australian Classification of Health Interventions for procedures. The grouping algorithm is based on AR DRG version 5.2, which assigns cases to 671 DRG classes. Mandatory grouping variables include patient age; date of admission and discharge; principal diagnosis; additional diagnosis; interventions; discharge status and birth weight at admission for newborns.

Hospital DRG data analysis included activity expressed as a total number of cases and their DRGs grouping; cases complexity distribution; casemix index (CMI); and hospital cost per DRG weighted case. Results were interpreted with the knowledge of some of the shortcomings of the DRG system implementation, such as coding accuracy and inconsistencies in the development of DRG grouping algorithms. Financial data were used to ascertain hospital financial status by relating their inpatient funding to their expenditure.

## RESULTS

Hospitals obtain funding from a number of sources. These income streams (Table 1) are categorized by CHIF as follows: payments by CHIF; supplementary funding from county and other government sources; extraordinary income, which is believed to include cyclical injections from the State Budget; and “other sources,” which include patient co-payments and income from other providers for services such as diagnostics.

**Table 1:** Hospital income categorized by the main income streams for the period 2009 to 2018*

Year	Payments by CHIF		Supplementary funding from county and other government sources		Extraordinary income		Other income		Total hospital Income
	HRK amount	% of total income	HRK amount	% of total income	HRK amount	% of total income	HRK amount	% of total income	HRK amount
2009	8 845 948 534	89	469 059 977	4.7	401 973 223	4.0	252 027 172	2.5	9 969 008 906
2010	8 678 484 772	88	462 986 876	4.7	492 395 395	5.0	234 489 048	2.4	9 868 356 091
2011	8 757 836 946	90	366 788 564	3.8	264 846 783	2.7	306 012 256	3.2	9 695 484 549
2012	8 636 917 996	87	660 072 646	6.7	369 242 616	3.7	252 939 447	2.6	9 919 172 705
2013	8 527 010 495	86	965 239 367	9.7	253 053 526	2.5	208 488 300	2.1	9 953 791 688
2014	7 536 497 216	73	1 947 526 985	18.9	619 713 745	6.0	223 485 976	2.2	10 327 223 922
2015	9 008 118 563	90	334 478 012	3.3	385 788 038	3.9	271 205 806	2.7	9 999 590 419
2016	9 539 845 674	91	306 528 098	2.9	345 032 574	3.3	302 042 343	2.9	10 493 448 689
2017	9 596 956 458	80	1 227 794 959	10.2	828 872 406	6.9	407 723 094	3.4	12 061 346 917
2018	10 321 208 439	83	683 547 259	5.5	936 328 736	7.5	495 019 114	4.0	12 436 103 548
Total/Average	89 448 825 093	85	7 424 022 743	7.1	4 897 247 042	4.7	2 953 432 556	2.8	104 723 527 434

*Source: Croatian Health Insurance Fund (CHIF).

On average, CHIF provides 85% of the total funding of the 33 studied public acute hospitals. This proportion, however, varied markedly over the period, from 73% in 2014 to 91% in 2016. In 2014 and 2017, the years in which the proportion of CHIF funding was lower than usual, the shortfall was more than compensated by increased supplementary funding from county and other government sources, as well as by extraordinary and other income. While the actual origins of this non-CHIF revenue, which oscillated between 9.1% of total revenue in 2016 to 27% in 2014, have not been reported, they likely include transfers from the State Budget and were applied to cover hospital debts as they accrued.

There was considerable year-to-year variation in the financial status of the hospital network as a whole (Table 2). In 2009, the hospital network had a surplus of HRK 15.8 million, while in 2018 it was operating at a deficit of HRK 808.6 million, with a total accumulated debt of HRK 1516 million.

**Table 2:** Financial status of the acute hospital system between 2009 and 2018*

Year	Hospital income from CHIF (HRK)	Total hospital income (HRK)	Total hospital expenditure (HRK)	Hospital network financial balance (HRK)	Running total of hospital network financial balance (HRK)	Financial balance as % of expenditure (HRK)	CHIF hospital income less total hospital expenditure (HRK)	Running total of shortfall in CHIF Hospital funding (HRK)
2009	8 806 021 710	9 969 008 906	9 953 225 081	15 783 825	15 783 825	0.20	-1 147 203 371	-1 147 203 371
2010	8 640 575 557	9 868 356 091	9 951 563 358	-83 207 267	-67 423 442	-0.80	-1 310 987 801	-2 458 191 172
2011	8 757 836 946	9 695 484 549	10 088 264 178	-392 779 629	-460 203 071	-3.90	-1 330 427 232	-3 788 618 404
2012	8 636 917 996	9 919 172 705	9 999 683 907	-80 511 202	-540 714 273	-0.80	-1 362 765 911	-5 151 384 315
2013	8 527 010 495	9 953 791 688	9 782 552 944	171 238 744	-369 475 529	1.80	-1 255 542 449	-6 406 926 764
2014	7 536 497 216	10 327 223 922	9 788 810 715	538 413 207	168 937 678	5.50	-2 252 313 499	-8 659 240 263
2015	9 008 118 563	9 999 590 419	10 452 148 581	-452 558 162	-283 620 484	-4.30	-1 444 030 018	-10 103 270 281
2016	9 539 845 674	10 493 448 689	11 151 276 799	-657 828 110	-941 448 594	-5.90	-1 611 431 125	-11 714 701 406
2017	9 596 956 458	12 061 346 917	11 827 143 579	234 203 338	-707 245 256	2.00	-2 230 187 121	-13 944 888 527
2018	10 321 208 439	12 436 103 548	13 244 724 721	-808 621 173	-1 515 866 429	-6.10	-2 923 516 282	-16 868 404 809

*Source: Croatian Health Insurance Fund (CHIF) data and authors’ calculation.

The gap between income and expenditure across the hospital network increased during the study period. In 2009, the CHIF funding shortfall was HRK 1147 million, or 11% of total hospital expenditure, while in 2018 the funding gap was HRK 2923 million, or 22% of total hospital expenditure. Moreover, if the hospital network had relied exclusively on CHIF revenue, without top-up funding from other sources, the acute hospital debt in 2018 would have been HRK 16 868 million.

**Figure 1:**
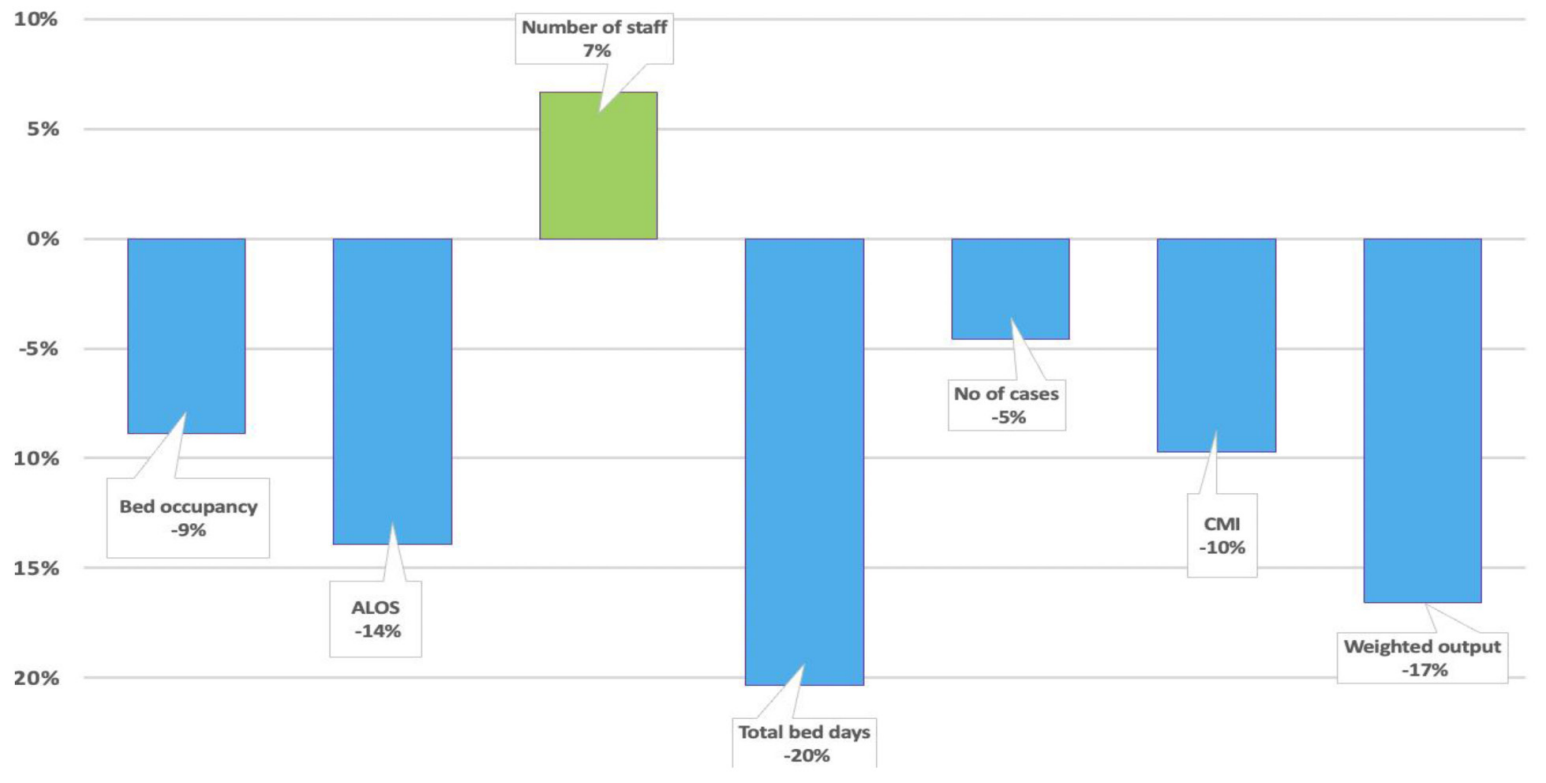
Percentage changes in hospital activity and resourcing between 2009 and 2018. ALOS – average length of stay; CMI – casemix index.

There was little sign of improvement in hospital efficiency. While on one hand, there was a reduction in average length of stay (ALOS), on the other hand, there was a reduction in hospital inpatient activity, but an increase in overall staffing levels (Figure 1, Supplementary Table 1(Supplementary Table 1)).

While ALOS across the network decreased from 7 days to 6 days, it varied considerably between hospitals. In 2018 for example, ALOS of non-specialist tertiary hospitals varied between 7.3 and 5.5 days, while in secondary hospitals, the range was 6.6 to 5.2 days. Reduction in ALOS was accompanied by a 12% decrease in acute bed numbers across the network. Some 70% of this reduction (1377 beds) occurred in secondary-level hospitals, reducing their acute bed capacity by 20%. Despite the reduction in bed numbers and ALOS, bed occupancy rate decreased from 79% in 2009 to 72% in 2018. This decline was most evident among tertiary-level hospitals, where it dropped by 16%, from an occupancy rate of 86% in 2009 to 72% in 2018.

The study period also witnessed a 5% reduction in the number of admissions. In 2009, 641 888 admitted cases were recorded and activity fluctuated around this number until 2016, when it began to decrease, ending up at 612 369 cases in 2018. Much of this reduction occurred in secondary-level hospitals, which experienced 15% decrease in admissions, from 287 572 cases in 2009 to 244 444 cases in 2018. The reason for the decrease in admissions is not known, although it is expected that a contributing factor may be an increase in same-day procedures, which in the Croatian system are not counted as hospital admissions. While this change in medical practice may have had some impact, it is unlikely to explain the 15% decrease in admissions in secondary hospitals when compared with a 4% increase in tertiary hospitals.

Notwithstanding the reduction in the number of beds, ALOS, and inpatient activity, hospital staff numbers increased by 7%, from 40 637 to 43 354, and the staff-bed ratio increased from 2.6 staff per bed in 2009 to 3.1 staff per bed in 2018. Significantly, the increase in staffing levels was most noted in secondary-level hospitals, which increased their staff-bed ratio by 35%, from 2.3 to 3.1, despite a decrease in their activity and lesser complexity of their case load.

The hospital casemix index (CMI) is an indicator of the average case complexity as measured by the DRG system. The average hospital CMI declined over the study period from 1.03 in 2009 to 0.93 in 2018. The CMI considerably varied between hospitals. In 2018 for example, the average CMI in non-specialist tertiary hospitals was 1.17 (with a variance of 1.27-0.92) compared with 0.84 for secondary-level hospitals (with variance of 1.03-0.70) (Figure 1).

The CMI is used in the calculation of the DRG-weighted inpatient output for each hospital. The total DRG-weighted output of all hospitals decreased by 17%, from 714 435 to 596 078 over the study period (Table 3). In tertiary-level hospitals, the weighted output decreased by 13%, from 448 502 to 389 845 and in secondary-level hospitals it decreased by 22%, from 265 933 to 206 233. This finding is not altogether unexpected as the period saw a decrease in both the number of admissions, and in case complexity measured by the CMI.

**Table 3:** Hospital efficiency as indicated by average cost per diagnosis-related group weighted case between 2016 and 2018*^†^

	Average cost per weighted case		
Average cost of case	2016	2017	2018
Tertiary hospitals	10 672	11 421	12 144
Secondary hospitals	12 407	13 375	14 908
Acute hospital network	11 829	12 724	13 987

*Source: Data from Croatian Health Insurance Fund (CHIF) and authors’ calculations.

†The average cost per weighted case was calculated with the assumption that the DRG inpatient expenditure was 70% of the total hospital expenditure after deducting expenditure reimbursed by CHIF for expensive drugs, transplants, and interventional cardiology and neurology; the resulting DRG inpatient expenditure was divided by the DRG weighted output to calculate the average cost per weighted case.

The cost per DRG-weighted case is an indicator of hospitals’ technical efficiencies and was calculated by dividing the DRG-weighted output into hospital’s expenditure on that output. The trend of the cost per DRG-weighted case for the hospital network over a 3-year period from 2016 to 2018 increased by 18%, from HRK 11 828 to HRK 13 987 (Table 3), indicating a loss in cost efficiency across the hospital network (Supplementary Table 2(Supplementary Table 2)).

The analysis revealed considerable disparity in costs of production between hospitals (Table 3). In 2018 for example, secondary level hospitals had an average cost per weighted case of HRK 14 908 and by that measure were less efficient than tertiary-level hospitals, which operated at an average cost per weighted case of HRK 12 144. Notably, there was also a marked variance in the average cost per weighted case among secondary-level hospitals, which provide a comparable set of services. Costs ranged between HRK 10 975 and HRK 23 066 – which means that the most efficient secondary hospital in the network was producing at less than 50% of the cost of the least efficient.

**Figure 2:**
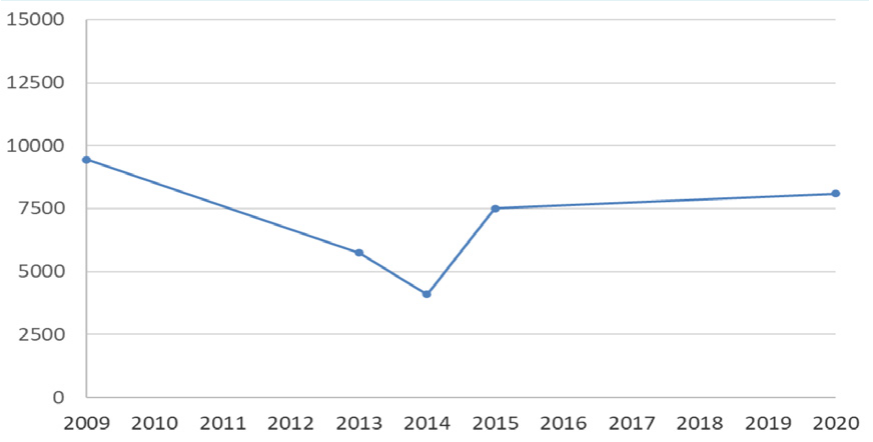
Changes in drug related grouping base price set by Croatian Health Insurance Fund between 2009 and 2020. Source: Data from CHIF.

The DRG prices are the function of the base price and price-weights for each DRG class. In Croatia, the base price is set by CHIF, which varied it over the study period. The base price oscillated between HRK 9400 in 2009 and HRK 4100 in 2014 (Figure 2), which is incidentally the year in which CHIF funded only 73% of the total hospital income (Table 1).

For instance, in 2018 the CHIF base price was set at HRK 7500 (Table 3). This was some 53% of the average cost per weighted case in that year, which was HRK 13 987 and which by definition should relate to the base price. In other words, in 2018 CHIF paid hospitals at a price that funded only 53% of their average cost of inpatient cases.

## DISCUSSION

The key finding of the study is that the cost efficiency of Croatian hospitals over a 10-year period did not noticeably improve since the introduction of DRGs. This is particularly so if judged by increasing inputs such as hospital expenditures and staffing level, and decreased outputs such as number of inpatient cases and DRG weighted activity. To the contrary, there is evidence that secondary hospitals became less efficient over the period based on these dimensions.

One of the most commonly debated dimensions of health system performance is that of health system efficiency (6). Inefficiency in any part of the health systems can lead to undesirable consequences, including inferior outcomes for patients. The World Health Report (2010) observed that of the nine leading contributors to health system inefficiency, at least five were related to hospital care and include wasteful procedures where they are unnecessary or duplicated; poor outcomes due to shortcomings in the quality of care; discrepancy between hospital size and their purpose; unwarranted hospital admissions; and longer than necessary hospital stays (7). Given that in most countries hospital care consumes more than 40% of the health budget (8), the efficiency of the hospital sector is a significant factor in the efficiency of the health system as whole. These DRG observations provide an invaluable opportunity to observe performance and enable hospitals and clinicians to set and manage improvement objectives.

A stated purpose of DRG-based hospital funding reforms is to enable hospitals and clinical teams to improve hospital system efficiency (9-11). While Croatia has made progress in assessing hospital activity using DRG classifications and in introducing the concept of activity-based funding into the hospital payment formula, it is difficult to demonstrate that the reform has meaningfully affected the efficiency of the hospital system. Indeed, if the purpose of hospital funding reforms is to enable and incentivize hospitals to improve their efficiency, the Croatian hospital funding framework and payment model is set up for this not to happen.

While a number of factors may have contributed to this outcome, the key reason is that the introduction of the DRG classification and performance measurement system was not accompanied by complementary changes to the hospital funding and payment mechanism. This oversight hindered the creation of incentives that would motivate hospital management to improve the efficiency of their institutions (12,13).

To be successful, a hospital payment reform strategy calls for the collaboration of key stakeholders, and the legitimacy of the payment system requires a budgeting process that reflects hospitals’ actual funding needs (14-16). While CHIF asserts that hospital budget setting is based on the business plans submitted by hospitals, its actual budgeting process can be seen as an annual reckoning based on the availability of funds. The effect of this approach is that CHIF ends up underpaying hospitals rather than being selective and rewarding those hospital teams that are demonstrably more efficient. Moreover, CHIF’s rather arbitrary approach to DRG price setting results in hospitals being paid at a price that is significantly below their costs of production. The result is that the more inpatient services that hospitals provide in order to meet CHIF’s activity targets, the greater is the debt that they generate. It can be argued, therefore, that CHIF’s approach to reimbursing hospitals creates perverse incentives that, if anything, discourage efficiency.

Therefore, while being the key actor, CHIF is yet to embrace its institutional responsibility as purchaser, as under the current system of governance, it is aware that it will not be censured for underpaying hospitals. Furthermore, hospitals operate in the same system with the knowledge that they will face no real consequences when they generate debt. In this rather dysfunctional environment, CHIF has little leverage in its purchaser/provider relationship with hospitals as both parties are aware that at the end, debts generated by hospitals will be covered by other public sources such as the MOH, local governments, and the State Budget.

If CHIF pays hospitals as much as it can afford, the question arises if CHIF is in fact underfunded. CHIF's main source of revenue comprises two income streams. The first is mandatory contributions from the employed members of the community, and the second takes the form of State Budget contributions made on behalf the exempted category of the population as stipulated by the Law on Mandatory Health Insurance. It would appear, however, that the State Budget does not meet the full costs of health care of people in the exempted category, with the result that those in the workforce cross-subsidize the care of those who are not. In 2016 for example, mandatory contribution revenue from those in the workforce, who form 34% of the population, amounted to HRK 23 billion, while the State Budget transfer for the exempted category was only HRK 2.5 billion (17).

While the aim of this article was to address issues of funding, it should also be mentioned that the gain of efficiency benefits from the DRG system funding of inpatient care was impeded by technical missteps. These included CHIF's technically flawed changes to the DRG classification system and price-weights. For instance, the unexplained change to DRG grouping algorithm in 2016 resulted in a 30% reduction in hospital reporting of the most complex cases (Patient Clinical Complexity Level A) and significantly undervalued hospitals' DRG activity. Moreover, insufficient effort was put into coder training and audit systems, which resulted in anomalies in some DRG data (18-20).

While the study makes use of comprehensive DRG data for all 33 acute hospitals in Croatia, there are discrepancies in some of the activity data most likely due to shortcomings in DRG coding practice. The nature of these inaccuracies is understood and they not affect the key findings of the article.

Consideration was also given to the potential effects of inflation. Since the study in the main compares funding and expenditure relativities within the 10-year study period rather than trends of actual monetary amounts, it is expected that any adjustment for inflation will not change the conclusions of the analysis.

While the study indicated that the Croatian health system can access the necessary funding to meet the current costs of hospital production, it exposed significant shortcomings in hospital funding flows as well as the payment model. The key actors – MOH, CHIF, and the State Budget – failed to coordinate their funding. Consideration may be given to amalgamating available funds into a single pool that is used to reimburse hospitals at appropriate levels within a payment model providing incentives for efficiency gains as well as improvements in the quality of care.

Importantly, these problems have likely arisen from shortcomings in the governance of the reforms and a lack of clarity in the allocation of accountabilities among the key stakeholders. The governance dimensions that may be addressed in this instance include coherent decision-making structures; consistency of purpose; stakeholder participation; regulation and supervision of interventions; and transparency and information (21).

Croatia can also learn from the experience of countries that effectively implemented a DRG activity-based payment model, such as Australia, Germany, Ireland, and the Scandinavian countries (22-25). The main lesson from these countries is that effective health financing reform calls for an integrated institutional approach that involves a link between purchasing and funding, expenditure control, independent price setting, and on-going technical development and maintenance.

Croatia should build on its own experience and experiences of other countries and develop technical capacity and create a governance framework. This framework should connect the efforts of key actors in an enabling process where the roles and responsibilities of key actors are defined and where they work in concert to implement agreed to reform policies (26). As in other countries, such effort may be coordinated by an independent hospital payment agency that would support CHIF in operating the hospital funding pool and payment system; amending classification in response to changes in health service technology; conducting costing studies to inform all actors on the real costs of production; and advising on policies to support and incentivize hospital efficiency gains. Importantly, such an agency would provide hospitals with the confidence that the payment system is set up to pay hospitals fairly for the work that they do, while at the same time inspiring them to respond by working to make gains in both efficiency and effectiveness.

To facilitate this, it is expected that Croatia will benefit from further research in the form of a descriptive study that uses qualitative methods to investigate the experience of other countries in the implementation of the DRG system. The product of such research would be an evidence-based plan for systemic enhancements that work collectively to improve the efficiency of the hospital sector.
