# Supplementary material for: The effects of diagnosis-related groups payment on efficiency of the hospital health care in Croatia
Source: Croat Med J. 2021 Dec;62(6):561–8. doi: 10.3325/cmj.2021.62.561 (PMC8771233; doi:10.3325/cmj.2021.62.561)
Supplement: Supplementary Table 1 [file CroatMedJ_62_s002.pdf]

Supplementary Table 1

		Acute Beds	Bed occupancy	ALOS	Number of staff	Total bed days	No of cases	staff/bed ratio	CMI	Weighted output (O)
**2009**	TOTAL TERTIARY	8,940	86%	7.92	24,546	2,787,035	354,316	2.75	1.30	448,502
	TOTAL SECONDARY	6,898	75%	6.49	16,091	1,880,198	287,572	2.33	0.90	265,933
	**TOTAL/AVERAGE**	**15,838**	**79%**	**6.97**	**40,637**	**4,667,233**	**641,888**	**2.57**	**1.03**	**714,435**
**2018**	TOTAL TERTIARY	8,371	72%	6.45	26,115	2,272,889	367,925	3.12	1.17	389,845
	TOTAL SECONDARY	5,521	71%	5.85	17,239	1,444,609	244,444	3.12	0.84	206,233
	**TOTAL/AVERAGE**	**13,892**	**72%**	**6.00**	**43,354**	**3,717,498**	**612,369**	**3.12**	**0.93**	**596,078**
	**Precentage Change**	**-12%**	**-9%**	**-14%**	**7%**	**-20%**	**-5%**		**10%**	**-17%**
