# Supplementary material for: The effects of diagnosis-related groups payment on efficiency of the hospital health care in Croatia
Source: Croat Med J. 2021 Dec;62(6):561–8. doi: 10.3325/cmj.2021.62.561 (PMC8771233; doi:10.3325/cmj.2021.62.561)
Supplement: Supplementary Table 2 [file CroatMedJ_62_s003.pdf]

Supplementary Table 2

HOSPITAL	Average Cost Per Weighted Case 2016	Average Cost Per Weighted Case 2017	Average Cost Per Weighted Case 2018
UHC SPLIT	10,512	12,396	12,496
UHC ZAGREB	12,631	13,604	15,473
UHC OSIJEK	11,325	10,926	11,972
UHC RIJEKA	9,387	10,983	11,987
UH "Sisters of Mercy" ZAGREB + Children's Hospital Zag	10,632	10,654	11,427
CH DUBRAVA	9,993	10,727	11,030
CH MERKUR	8,807	8,750	9,464
ORTHOPAEDIC CLINIC LOVRAN	5,509	6,167	6,292
UH for infectious diseases "Dr. Fran Mihaljević" ZAGREB	17,756	19,209	18,250
CH Holy Ghost	10,169	10,799	13,053
**Average cost of case for tertiary hospitals**	**10,672**	**11,421**	**12,144**
GH GOSPIĆ	11,118	12,461	12,373
GVH CROATIAN PRIDE KNIN	15,984	18,449	17,932
GH VUKOVAR	19,459	19,158	23,066
GH BJELOVAR	11,440	11,471	11,852
COUNTY HOSPITAL ČAKOVEC	9,905	9,851	11,223
GH DUBROVNIK	11,918	14,051	16,758
GH KARLOVAC	11,550	10,951	13,539
GH KOPRIVNICA	10,096	10,042	11,989
GH NAŠICE	11,020	11,932	13,067
GH OGULIN	12,728	14,515	15,914
GH POŽEGA + GH PAKRAC	11,562	12,081	11,235
GH PULA	16,109	18,438	22,649
GH SISAK	12,547	13,566	14,799
GH SL. BROD + GH NOVA GRADIŠKA	10,762	11,929	12,923
GH ŠIBENIK	14,141	15,176	18,001
GH VARAŽDIN	12,203	13,496	15,729
GH VINKOVCI	13,273	12,924	14,666
GH VIROVITICA	11,486	12,348	15,136
GH ZABOK	10,968	12,722	14,345
GH ZADAR	9,871	11,934	10,975
**Average cost of case for secondary hospitals**	**12,407**	**13,375**	**14,908**
**Average cost of case for the acute hospital network**	**11,829**	**12,724**	**13,987**
